# Clinical implementation of RNA sequencing for Mendelian disease diagnostics

**DOI:** 10.1186/s13073-022-01019-9

**Published:** 2022-04-05

**Authors:** Vicente A. Yépez, Mirjana Gusic, Robert Kopajtich, Christian Mertes, Nicholas H. Smith, Charlotte L. Alston, Rui Ban, Skadi Beblo, Riccardo Berutti, Holger Blessing, Elżbieta Ciara, Felix Distelmaier, Peter Freisinger, Johannes Häberle, Susan J. Hayflick, Maja Hempel, Yulia S. Itkis, Yoshihito Kishita, Thomas Klopstock, Tatiana D. Krylova, Costanza Lamperti, Dominic Lenz, Christine Makowski, Signe Mosegaard, Michaela F. Müller, Gerard Muñoz-Pujol, Agnieszka Nadel, Akira Ohtake, Yasushi Okazaki, Elena Procopio, Thomas Schwarzmayr, Joél Smet, Christian Staufner, Sarah L. Stenton, Tim M. Strom, Caterina Terrile, Frederic Tort, Rudy Van Coster, Arnaud Vanlander, Matias Wagner, Manting Xu, Fang Fang, Daniele Ghezzi, Johannes A. Mayr, Dorota Piekutowska-Abramczuk, Antonia Ribes, Agnès Rötig, Robert W. Taylor, Saskia B. Wortmann, Kei Murayama, Thomas Meitinger, Julien Gagneur, Holger Prokisch

**Affiliations:** 1 Institute of Human Genetics, School of Medicine, Technical University of Munich, Munich, Germany; 2 Department of Informatics, Technical University of Munich, Garching, Germany; 3 Quantitative Biosciences Munich, Department of Biochemistry, Ludwig-Maximilians-Universität, Munich, Germany; 4 Institute of Neurogenomics, Helmholtz Zentrum München, Neuherberg, Germany; 5 DZHK (German Centre for Cardiovascular Research), partner site Munich Heart Alliance, Munich, Germany; 6 Wellcome Centre for Mitochondrial Research, Translational and Clinical Research Institute, Faculty of Medical Sciences, Newcastle University, Newcastle upon Tyne, NE2 4HH UK; 7 NHS Highly Specialised Services for Rare Mitochondrial Disorders, Royal Victoria Infirmary, Newcastle upon Tyne Hospitals NHS Foundation Trust, Queen Victoria Road, Newcastle upon Tyne, NE1 4LP UK; 8 Department of Pediatric Neurology, Beijing Children’s Hospital, Capital Medical University, National Center for Children’s Health, Beijing, China; 9 Department of Women and Child Health, Hospital for Children and Adolescents, Center for Pediatric Research Leipzig (CPL), Center for Rare Diseases, University Hospitals, University of Leipzig, Leipzig, Germany; 10 Department for Inborn Metabolic Diseases, Children’s and Adolescents’ Hospital, University of Erlangen-Nürnberg, Erlangen, Germany; 11 Department of Medical Genetics, Children’s Memorial Health Institute, Warsaw, Poland; 12 Department of General Pediatrics, Neonatology and Pediatric Cardiology, Heinrich-Heine-University, Düsseldorf, Germany; 13 Department of Pediatrics, Klinikum Reutlingen, Reutlingen, Germany; 14 University Children’s Hospital Zurich and Children’s Research Centre, Zürich, Switzerland; 15 Department of Molecular and Medical Genetics, Oregon Health & Science University, Portland, USA; 16 Institute of Human Genetics, University Medical Center Hamburg-Eppendorf, Hamburg, Germany; 17 Research Centre for Medical Genetics, Moscow, Russia; 18 Diagnostics and Therapeutics of Intractable Diseases, Intractable Disease Research Center, Juntendo University, Graduate School of Medicine, Tokyo, Japan; 19 Department of Life Science, Faculty of Science and Engineering, Kindai University, Osaka, Japan; 20 Department of Neurology, Friedrich-Baur-Institute, University Hospital, Ludwig-Maximilians-Universität, Munich, Germany; 21 German Center for Neurodegenerative Diseases (DZNE), Munich, Germany; 22 Munich Cluster for Systems Neurology (SyNergy), Munich, Germany; 23 Unit of Medical Genetics and Neurogenetics, Fondazione IRCCS (Istituto di Ricovero e Cura a Carattere Scientifico) Istituto Neurologico Carlo Besta, Milan, Italy; 24 Division of Neuropediatrics and Pediatric Metabolic Medicine, Center for Pediatric and Adolescent Medicine, University Hospital Heidelberg, Heidelberg, Germany; 25 Department of Pediatrics, Technical University of Munich, Munich, Germany; 26 Research Unit for Molecular Medicine, Department of Clinical Medicine, Aarhus University, Aarhus, Denmark; 27 Section of Inborn Errors of Metabolism-IBC, Department of Biochemistry and Molecular Genetics, Hospital Clínic, IDIBAPS, CIBERER, Barcelona, Spain; 28 Department of Pediatrics & Clinical Genomics, Faculty of Medicine, Saitama Medical University, Saitama, Japan; 29 Center for Intractable Diseases, Saitama Medical University Hospital, Saitama, Japan; 30 Inborn Metabolic and Muscular Disorders Unit, Anna Meyer Children Hospital, Florence, Italy; 31 Department of Pediatric Neurology and Metabolism, Ghent University Hospital, Ghent, Belgium; 32 Department of Pathophysiology and Transplantation, University of Milan, Milan, Italy; 33 University Children’s Hospital, Paracelsus Medical University Salzburg, Salzburg, Austria; 34 Université de Paris, Institut Imagine, INSERM UMR 1163, Paris, France; 35 Amalia Children’s Hospital, Radboudumc Nijmegen, Nijmegen, The Netherlands; 36 Department of Metabolism, Chiba Children’s Hospital, Chiba, Japan; 37 Institute of Computational Biology, Helmholtz Zentrum München, Neuherberg, Germany

**Keywords:** RNA-seq, Genetic diagnostics, Mendelian diseases

## Abstract

**Background:**

Lack of functional evidence hampers variant interpretation, leaving a large proportion of individuals with a suspected Mendelian disorder without genetic diagnosis after whole genome or whole exome sequencing (WES). Research studies advocate to further sequence transcriptomes to directly and systematically probe gene expression defects. However, collection of additional biopsies and establishment of lab workflows, analytical pipelines, and defined concepts in clinical interpretation of aberrant gene expression are still needed for adopting RNA sequencing (RNA-seq) in routine diagnostics.

**Methods:**

We implemented an automated RNA-seq protocol and a computational workflow with which we analyzed skin fibroblasts of 303 individuals with a suspected mitochondrial disease that previously underwent WES. We also assessed through simulations how aberrant expression and mono-allelic expression tests depend on RNA-seq coverage.

**Results:**

We detected on average 12,500 genes per sample including around 60% of all disease genes—a coverage substantially higher than with whole blood, supporting the use of skin biopsies. We prioritized genes demonstrating aberrant expression, aberrant splicing, or mono-allelic expression. The pipeline required less than 1 week from sample preparation to result reporting and provided a median of eight disease-associated genes per patient for inspection. A genetic diagnosis was established for 16% of the 205 WES-inconclusive cases. Detection of aberrant expression was a major contributor to diagnosis including instances of 50% reduction, which, together with mono-allelic expression, allowed for the diagnosis of dominant disorders caused by haploinsufficiency. Moreover, calling aberrant splicing and variants from RNA-seq data enabled detecting and validating splice-disrupting variants, of which the majority fell outside WES-covered regions.

**Conclusion:**

Together, these results show that streamlined experimental and computational processes can accelerate the implementation of RNA-seq in routine diagnostics.

**Supplementary Information:**

The online version contains supplementary material available at 10.1186/s13073-022-01019-9.

## Background

It is estimated that at least 3.5–6% of the human population is affected by a rare disease [1]. Presumably, ~80% of rare diseases have a genetic cause [2]. Although not necessarily providing the cure, establishing the correct and timely diagnosis of a Mendelian disease can improve disease management, provide prognostic information, and inform genetic counseling [3–5]. Clinical implementation of next-generation sequencing, especially whole exome sequencing (WES), revolutionized genetic diagnostics of individuals suspected of having a Mendelian disorder by improving diagnostic yield and accelerating the discovery of novel disease genes [6, 7]. Nevertheless, the diagnostic yield of WES analysis rarely exceeds 50% and hence leaves the majority of patients without a genetic diagnosis [8–12]. Inconclusive WES can be partially attributed to the challenges concerning variant detection, prioritization, and interpretation. Although whole genome sequencing (WGS) allows, in principle, the detection of all genomic variants, its clinical implementation has reported similar diagnostic rates to those of WES [13, 14]. This indicates that variant prioritization and interpretation are the main challenges in genetic diagnostics [15].

So far, variants predicted to have potentially large effects on protein function are limited to large copy number variations, loss-of-function variants such as frameshift, start loss, stop gain, and stop loss, and variants altering splice acceptor or donor dinucleotides [16]. However, it has been suggested that up to 30% of pathogenic variants fall within non-coding regions [17, 18]. Moreover, multiplex splicing assays showed that splicing-disturbing variants include about 10% of pathogenic exonic variants and are difficult to predict [19, 20]. Although many *in silico* tools have been developed to predict the effect of a variant on transcription, splicing, or RNA stability, their accuracy remains too low to establish a firm diagnosis. Without the necessary functional validation using either a minigene or patient biopsy material, splice region and non-coding variants remain as variants of uncertain significance (VUS [21]).

By directly probing transcript abundance and sequence on a transcriptome-wide basis, RNA-seq allows systematic identification of aberrant transcript events, defined as genes expressed at aberrant levels, aberrantly spliced genes, and mono-allelically expressed (MAE) rare variants. Detection of such events enables validation of VUS potentially affecting the transcript, re-interpretation of VUS when linked to an aberrant transcript event, and discovery of pathogenic variants not covered by WES. A recent study concluded that up to 31% of splicing VUSs could reach either a likely pathogenic or likely benign classification from RNA-seq analysis [22]. The application of RNA-seq has increased diagnostic rates by 8–36% across a variety of rare disorders and selected cohorts of up to approximately one hundred affected individuals [23–28]. Besides increasing the diagnostic yield, RNA-seq can improve the understanding of the molecular pathomechanism of the variant(s) and basic genetic mechanisms. While these initial studies are promising, routine clinical implementation of RNA-seq requires robust and efficient computational workflows, establishment of quality controls, and adequate RNA source material and sequencing depth.

**Fig. 1:**
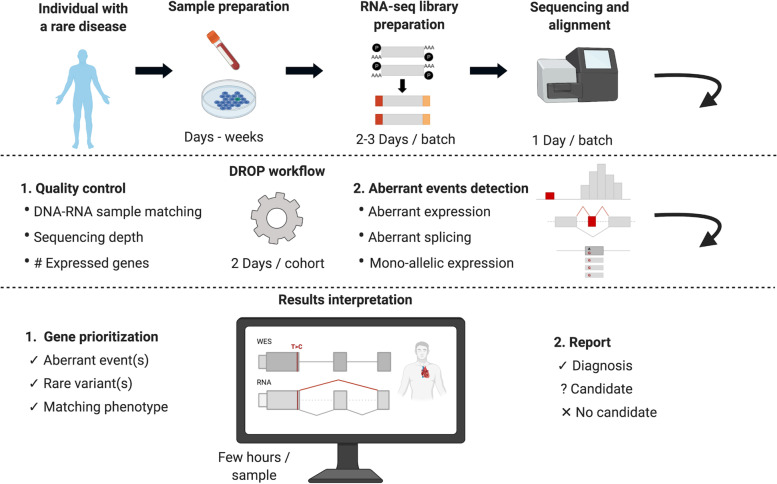
Experimental design of an RNA-seq based diagnostic study. First, individuals suspected of a Mendelian disorder are recruited for DNA sequencing. In addition, patient biopsy material is collected during the routine medical examination and prepared for RNA extraction. The sample preparation process can take from hours for biopsies to weeks for establishing a cell culture. RNA sequencing is then performed followed by alignment and quality control. The generated data go through DROP which consists of quality control steps and detection of aberrant RNA expression events. The results are then interpreted by sample, including the association of aberrant RNA expression events with rare variant(s) and the function of affected genes with the patient phenotype, which can lead to new diagnoses or candidates. Experience-based estimated durations are provided for each step

Here, we report on our experience on the implementation of RNA-seq into clinical diagnostics using patient-derived skin fibroblasts (Fig. 1). We demonstrate the application of our validated computational workflow, DROP [29], which integrates preprocessing and quality control steps, as well as modules for detecting aberrant expression, aberrant splicing, and MAE (Fig. 1), and to which we have added a new module for RNA-seq-based variant calling. We apply this workflow to a compendium of WES and RNA-seq samples of 303 individuals suspected of having a mitochondrial or another Mendelian disease, the largest such dataset to date. For each type of aberrant event, we examine their genetic background and provide diagnostic guidance through case studies. While our analysis is based solely on fibroblast-derived material and with the majority of individuals suspected of having a mitochondrial disorder, our study also addresses the value of other clinically accessible tissues for the diagnosis of Mendelian disorders.

## Methods

### Compendium

A total of 303 individuals with a suspected Mendelian disorder were recruited, out of which 263 were clinically suspected to suffer from a mitochondrial disease (Additional file 1: Table S1). The compendium includes 70 individuals from the Kremer et al. study [23], 152 individuals from a multi-omics study [30], and 81 additional individuals recruited by the centers participating in this study. WES and RNA-seq were performed in all of them. Three cases further required WGS as they were candidates from RNA-seq, but no conclusive variants were found via WES or RNA-seq. For each individual, we report the International Classification of Diseases (ICD version 10) code and sex (170 male and 133 female, Additional file 1: Table S1).

### Cell culture

Primary fibroblast cell lines obtained from patient skin biopsy were cultured in high glucose DMEM (Life Technologies) supplemented with 10% FBS, 1% penicillin/streptomycin, and 200 μM uridine at 37 °C and 5% CO_2_. All fibroblast cell lines tested negative for mycoplasma contamination.

### Whole exome sequencing

DNA was isolated from peripheral blood leukocytes or skin-derived fibroblasts using DNeasy Blood & Tissue Kit (Qiagen, Hilden, Germany) according to the manufacturer’s protocol. DNA concentration was measured using the Qubit™ dsDNA BR Assay Kit. In total, 3 μg of DNA was used for library preparation. Exonic regions from human DNA samples were enriched with the SureSelect Human All Exon V5/V6 kits from Agilent (Agilent Technologies, Santa Clara, CA, USA) and sequenced as 100 bp paired-end runs on Illumina HiSeq2500 or HiSeq4000 platforms (Illumina, San Diego, CA, USA). Reads were aligned to the human reference genome (UCSC build hg19) using Burrows-Wheeler Aligner v0.7.5a [31]. Single-nucleotide variants, as well as small insertions and deletions (< 200 bp), were detected with SAMtools v0.1.19 [32] and GATK v3.8 [33].

### Whole genome sequencing

One WGS library was established using a MGIEasy DNA Library Prep Kit v1.1, according to the manufacturer’s protocol and generated DNA nanoballs. Sequencing was performed using 100-bp paired-end reads on a MGISEQ-2000 using MGISEQ-2000RS High-throughput Sequencing Set PE100 v3.0. The other two WGS libraries were prepared with the TruSeq DNA PCR-Free Kit (Illumina). DNA was fragmented to an average length of 350 bp by sonication. Libraries were validated according to standard procedures and sequenced via 150 bp paired-end on a NovaSeq 6000 platform. After removing adapter sequences and low-quality reads by Trimmomatic v0.39 [34], reads were aligned and variants were called using the same procedures described in the previous subsection.

### Variant annotation and handling

Variants were annotated for consequence, location, minor allele frequencies (from the 1000 Genomes Project [35] and gnomAD [36] cohorts), and deleteriousness scores using the R interface to the Ensembl Variant Effect Predictor (VEP) v1.32.0 [16, 37]. For variants that fell on multiple transcripts and had therefore multiple predicted consequences, the one with the highest predicted impact was selected [38]. We considered a variant to be rare if the maximum minor allele frequency across both cohorts was lower than 0.001 and the frequency of the variant in our cohort was lower than 0.01. Variants are reported using the Human Genome Variation Society (HGVS) recommendations [39].

In order to detect whether a genomic position is expressed, we computed the RNA coverage using the coverage function from the GenomicAlignments R package v1.26.0 [40]. We defined a position to be expressed if the mean coverage across all samples was greater or equal to 10 reads.

### RNA sequencing

RNA was isolated from the patient-derived skin fibroblasts with the RNeasy mini kit (Qiagen, Hilden, Germany) according to the manufacturer’s protocol. RNA integrity number (RIN) was determined using the Agilent 2100 BioAnalyzer (RNA 6000 Nano Kit, Agilent Technologies, Santa Clara, CA, USA). Non-strand-specific RNA-seq was performed in 101 samples as previously described [23]. The rest of the RNA samples were sequenced strand-specifically, where library preparation was performed according to the TruSeq Stranded mRNA Sample Prep LS Protocol (Illumina, San Diego, CA, USA). Specifically, 1 μg of RNA was purified using poly-T oligo-attached magnetic beads and fragmented. The RNA fragments were reverse transcribed with the First Strand Synthesis Act D mix. The second-strand cDNA was generated with Second Strand Marking Mix that ensures strand specificity by replacing dTTP with dUTP. The resulting double-stranded cDNA was subjected to end repair, A-tailing, adaptor ligation, and library enrichment. The quality and quantity of the RNA libraries were assessed with the Agilent 2100 BioAnalyzer and the Quant-iT PicoGreen dsDNA Assay Kit (Life Technologies, Carlsbad, CA, USA). RNA libraries were sequenced as 100 bp paired-end runs on Illumina HiSeq2500 or HiSeq4000 platforms. Reads from RNA-seq were demultiplexed and then mapped with STAR v2.7.0a to the hg19 genome assembly, with default parameters plus setting the twopassMode to “Basic” to detect novel splice junctions [41].

### Variant calling in RNA-seq data

Variants were called on RNA-seq data using GATK best practices for RNA-seq short variant discovery [33]. Variants with a ratio of quality to depth of coverage < 2 that were strand biased (Phred-scaled fisher exact score >30) or belonging to an SNP cluster (3 or more SNPs within a 35 bp window) were filtered out, as suggested by GATK. Furthermore, variants not contained in a repeat masked region (as defined by RepeatMasker v4.1.0 [42]) and with 3 or more reads supporting the alternative allele were prioritized. For the benchmark analysis, 210 RNA-seq samples derived from suprapubic skin of the GTEx project were used. Only genomic positions with an RNA coverage of at least 3 reads were considered.

### Quality control

Reads falling in exonic regions and with low quality were quantified using RNA-SeQC v2.4.2 [43]. DROP v1.0.3 was used to compute the total sequencing depth per sample, percentage of mapped reads, and the number of expressed genes [29]. DROP was also used to determine whether an RNA-seq sample matches its annotated DNA sample. A cutoff of 0.7 distinctly separated the matching with the non-matching DNA-RNA pairs.

### Detection of aberrant expression

Detection of aberrant expression was fully based on DROP v1.0.3 [29]. We used as reference genome the GRCh37 primary assembly, release 29, of the GENCODE project [44] which contains 60,829 genes. We used the summarizeOverlaps function from the GenomicAlignments [40] R package to count reads that are paired with mates from the opposite strands (singleEnd = FALSE). We only considered reads that fell completely within an exon or span two exons from the same gene via splicing (mode = intersectionStrict). Reads that overlapped more than one feature were assigned to each of those features instead of being removed (inter.feature = FALSE). Genes with a 95th percentile FPKM < 1 were considered to be not sufficiently expressed and filtered out.

Expression outliers were found using OUTRIDER [45], which uses a denoising autoencoder to control for latent effects and returns multiple-testing corrected *p*-values (FDR) for each gene and sample. Significant events were defined as those with a FDR ≤ 0.05. All aberrant events were further inspected using the Integrative Genome Viewer [46]. The OUTRIDER-corrected biological coefficient of variation (BCV) was computed per gene as 1/√𝜃, where 𝜃 is the fitted dispersion from the negative binomial distribution.

### Detection of aberrant splicing

Splicing outliers were obtained using the aberrant splicing DROP module based on FRASER [47], an annotation-free aberrant splicing detection algorithm. FRASER uses a denoising autoencoder to control for latent effects and estimates splice-site level and gene-level multiple-testing corrected *p*-values for percent spliced-ins and splicing efficiencies*.* Exon-exon and exon-intron junctions with < 20 reads in all samples and for which the total number of reads at the donor and acceptor splice site is 0 in more than 95% of the samples were filtered out. From the FRASER output, splicing outlier genes were defined as those with Holm’s adjusted *p*-value across junctions of the tested gene < 0.1. Outlier junctions are defined as those in splicing outlier genes, with an FDR < 0.1 and an effect size larger than 0.3, where the effect size is defined as the absolute difference between the observed and the predicted percent spliced-in |Δψ|, or between the observed and the predicted splicing efficiency |Δθ|.

### Detection of mono-allelic expression

For mono-allelic expression analysis, only heterozygous single-nucleotide variants from WES were considered. Reads assigned to each allele were counted using the ASEReadCounter function from GATK v4.0 [48]. Positions with less than 10 reads in total were filtered out. Afterward, the negative binomial test described in Kremer et al. [23] was performed. This fully corresponds to the MAE module of DROP v1.0.3. ANEVA-DOT was run using the ANEVADOT_test function from its R package and the provided pre-calculated genetic variations from fibroblasts from GTEx [49]. Significant variants were defined as those with FDR ≤ 0.05.

### Association of outlier genes with WES rare variants

Variants were grouped by predicted consequence in a similar way as done in Li et al. [50], but with some minor modifications. Specifically, the variant categorization was as follows: splice: splice acceptor, splice donor, splice region; frameshift: frameshift, UTR: 3′ UTR, 5′ UTR, start lost; non-coding: downstream, upstream, intron, regulatory region, intergenic; coding: coding, deletion, insertion, missense, stop lost; stop: stop gained; synonymous: synonymous, stop retained. Each sample-gene combination was categorized as overexpression, underexpression, or non-outlier according to the OUTRIDER results. Then, for each of them, we searched for a rare variant and assigned the variant’s consequence group to it. A Fisher’s exact test was performed for each variant group against each expression outlier class, thus obtaining a *p*-value. If rare variants from multiple groups were found on a sample-gene combination, the group with the lowest *p*-value (therefore highest association) was selected. Afterward, for each outlier class, the proportion of each group of rare variants was computed (e.g., # of underexpression outliers with a rare stop variant/total # of underexpression outliers). 95% confidence intervals were obtained from a binomial test for all proportions. The procedure was repeated in a similar way for splicing outliers caused by aberrant percent spliced-in. Only protein-coding genes were considered. Samples with more than 20 expression outlier genes were discarded for the expression analysis, and samples with more than 40 splicing outlier genes were discarded for the splicing analysis.

### Association of WES rare variants with outlier genes

All rare variants in expressed protein-coding genes in autosomal chromosomes were considered. For each sample, each rare homozygous variant was matched with the corresponding outlier class (overexpression, underexpression, or non-outlier) of the gene where it is located. Then, for each group of rare variants, the proportion of each outlier class was computed (e.g., # of rare stop variants in a gene that is an underexpression outlier/total # of rare stop variants). Stop and frameshift variants that were in expressed positions and not in the last exon were marked as potential PTVs. The procedure was repeated in a similar way by associating rare variants with splicing outliers, but splitting the “splice” category into “splice site” (which includes both donor and acceptor dinucleotides) and “splice region.”

MAE was tested on each rare heterozygous SNV in genes in autosomal chromosomes. Then, for each group of rare variants, the proportion of each MAE category (towards the reference or alternative allele, or none) was computed (e.g., # of rare stop SNVs with MAE of the alternative allele/total # of rare stop SNVs).

### Enrichment of gene classes

We performed pairwise logistic regression where the response variable is the outlier class and the predictor is the gene category. The odds ratio and 95% confidence interval were derived from the estimates and standard errors of the coefficients.

### GTEx dataset

This dataset consists of 7842 RNA-seq samples from 48 tissues of 543 assumed healthy individuals of the Genotype-Tissue Expression Project V6p [51]. The data were downloaded from the GTEx Portal on June 12, 2017, under accession number dbGaP: phs00424.v6.p1.

### Lists of genes

OMIM genes were downloaded from its portal (www.omim.org). Mitochondrial disease genes are our own expansion from the list shared in ref. [52] Hematology, neurology, and ophthalmology genes were extracted from ref. [25], neuromuscular genes were taken from ref. [26], and skeletal dysplasia genes from ref. [28]. Imprinted genes were taken from ref. [53]. LoF intolerant genes correspond to the genes with a loss-of-function observed/expected upper bound fraction < 0.35 from ref. [36].

## Results

### RNA-seq analysis workflow

Extending the study of Kremer and colleagues [23] to support routine diagnostic testing, we recruited 303 individuals suspected to be affected by a Mendelian disorder with fibroblasts cell lines available within an international collaboration and performed WES and RNA-seq on them (Additional file 2: Fig. S1, Methods). Almost all individuals (87%, 263 out of 303) were clinically suspected to suffer from a mitochondrial disease, presenting with a broad spectrum of clinical signs and symptoms. Mitochondrial disease represents an attractive class of rare disorders for the development and testing of systematic large-scale diagnostic screening approaches on account of significant clinical and genetic heterogeneity, with pathogenic variants described in more than 340 genes [52]. The study cohort consists of 106 WES-diagnosed cases used to establish a reference dataset of gene expression (some of which have been published as single-gene studies [4, 54–78]) and 197 cases that remained inconclusive after WES (Additional file 2: Fig. S1). A single RNA-seq assay was performed per individual at a median sequencing depth of 90 million reads (range 50–165 million reads, Additional file 2: Fig. S2A). A total of 101 samples were sequenced following a non-strand-specific protocol and 202 following a strand-specific one using automated protocols minimizing sample handling and allowing highly reproducible results (Methods). We provide the gene expression count matrices, as well as the privacy-preserving count matrices of split and unsplit reads overlapping annotated splice sites via Zenodo independently for the non-strand-specific (https://zenodo.org/record/4646823 [79]) and the strand-specific datasets (https://zenodo.org/record/4646827 [80]). These matrices can be integrated by external users through DROP [29].

After alignment, RNA-seq data were analyzed using the computational workflow DROP [29], which ensures reproducibility, robustness, and scalability (Fig. 1, Methods). All the samples had a high percentage of high-quality reads aligned (> 80% for all samples) and expressed more than 11,000 genes (Additional file 2: Fig. S2B-C). DROP also computes the percentage of matching DNA-RNA variants to control for sample mismatches, which allowed us to reassign ten RNA-seq samples to their corresponding DNA (Additional file 2: Fig. S3, Methods). Afterward, through DROP, we called aberrant expression, aberrant splicing, and MAE using the statistical methods OUTRIDER [45], FRASER [47], and a negative binomial test [23], respectively. This yielded a median of 25 aberrant genes per sample, including eight where variants have been reported to cause a Mendelian disease in humans (OMIM [81], Additional file 2: Fig. S4A-B). From RNA isolation until candidate identification via data analysis, the workflow has been streamlined by applying standardized protocols and semi-automated analysis pipelines which, in principle, allow to call outliers within 1 week (Fig. 1).

**Fig. 2:**
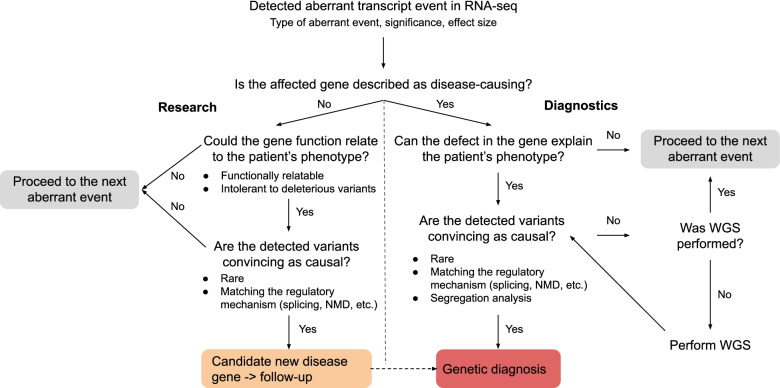
RNA-seq-based diagnostic flow chart. Flow diagram showing the diagnostic decision guideline after detecting a gene with an aberrant event in RNA-seq data. Identification of an aberrant event can lead to genetic diagnosis (diagnostic setting), lead to the discovery of a candidate new disease gene (research setting), or alternatively be of unlikely diagnostic significance, after which the next aberrant event is analyzed following the same path

Aberrant events involving known disease-associated genes were then inspected manually in a case-by-case fashion by comparing patient phenotype information with the phenotypes and mode of inheritance associated with the disease-associated gene, following the flow diagram shown in Fig. 2. For plausible candidate genes, we next inspected the sequencing data and searched for causative variants called by either WES or RNA-seq, and in some cases performed WGS followed by segregation analysis of the likely pathogenic variants. This procedure led to a genetic diagnosis of 32 unsolved cases, representing 16% (95%-CI 11–22%) of the WES-inconclusive cohort (see summarized case-by-case version in Table 1 and expanded one in Additional file 1: Table S2). Seven of the reported solved cases were previously published [23, 47, 82], and nine described in a companion manuscript [30]. Among the 46 causative variants in these 32 cases, 13 (28%) were already classified as pathogenic or likely pathogenic, 10 (22%) required functional validation, 11 (24%) were not prioritized during WES analysis, and 12 (26%) were not captured by WES. Three (25%) of the uncaptured group required WGS to identify the causative variant, while the other nine (75%) were detected using variant calling from RNA-seq (Table 1). In addition to the solved cases, we identified potential candidates in 12 cases: in 8 we identified a likely pathogenic change at the transcript level but have been unable to pinpoint the causative variant, and in 4 cases we identified aberrant expression and likely deleterious variants in candidate genes, representing likely novel disease genes (see Additional file 1: Table S3 for case-by-case description, Additional file 2: Fig. S4C). These candidate cases are currently being investigated in follow-up studies. Overall, the clinical interpretation of aberrant RNA phenotypes resulted in diagnosis for 16% of cases, including validation of suspected and non-suspected variants, and also discovery of WES-undetected pathogenic variants. In another 4% of cases, we detected likely pathogenic changes which need further follow-up studies. We did not find a specific pattern arising between the unsolved patients and the solved ones. In the following, we outline each screening step for the detection of aberrant events.

**Table 1:** Summary of cases diagnosed via RNA-seq. AE: aberrant expression, AS: aberrant splicing, MAE: mono-allelic expression, Var: intronic variant detected via RNA-seq. Variant coordinates and further details are provided in Additional file 1: Table S1

Index	Patient ID	Sex	Age range of onset	Primary symptoms	Genetic diagnosis	Variant RNA level	Variant class	RNA defects
**1**	**R62943**	F	Prenatal	Neurodevelopmental delay, 3-MGA	***C19orf70***	c.143del	Frameshift	AE, AS
					NM_205767.1	c.29+272G>C	Intronic	
**2**	**R98254**	F	Infant	Leigh syndrome, basal ganglia abnormality MRI, neurodevelopmental delay, intellectual disability, seizures, encephalopathy, brainstem abnormality MRI, complex I and IV defects	***MRPL38***	c.770C>G	Missense	AE
					NM_032478.3	c.-174_-148del	5′UTR deletion	
**3**	**R86287**	M	Infant	Hypotonia, cardiomyopathy, white matter abnormality MRI, elevated lactate, complex I and IV defects	***DARS2***	c.492+2T>C	Splice donor	AS
					NM_018122.4	c.228-12C>G; c.228-20T>C	Intronic multi-nucleotide variant (MNV)	
**4**	**R89912**	M	Infant	Leigh syndrome, basal ganglia abnormality MRI, neurodevelopmental delay, speech delay, intellectual disability, encephalopathy, hypotonia, nystagmus, brainstem abnormality MRI, elevated lactate, metabolic acidosis, complex I defect	***NFU1***	c.362T>C	Missense	AE, MAE
					NM_001002755.2	c.485-2588_545+1655del	Deletion	
**5**	**R19100**	M	Child	Myopathic facies, exercise intolerance, muscle weakness, motor, growth, speech and neurodevelopmental delay, intellectual disability, microcephaly, hypotonia, cardiomyopathy, dysmorphic features, ragged red fibers, elevated lactate	***SLC25A4***	c.598G>A	Splice region	AE
					NM_001151.3	c.598G>A	Splice region	
**6**	**R15264**	F	Infant	Muscle weakness, myopathy, muscular dystrophy, hypotonia	***TIMMDC1***	c.596+2146A>G	Intronic	AE, AS, Var
					NM_016589.3	c.596+2146A>G	Intronic	
**7**	**R36605**	M	Infant	Acute liver failure, hypotension of the muscles, hypertension of the limbs, intermittent deficiency of motor function of the pupil, delayed light reaction and nystagmus	***TWNK***	c.1302C>G	Synonymous	AE, AS
					NM_001163812.1	c.1302C>G	Synonymous	
**8**	**R61100**	F	Infant	Encephalopathy, respiratory distress	***NAXE***	c.292-12C>G	Intron	AE, AS
					NM_144772.2	c.292-12C>G	Intron	
**9**	**R77611**	F	Infant	Recurrent acute liver failure	***DLD***	c.685G>T	Missense	AE, MAE
					NM_000108.3			
**10**	**R16472**	M	Child	Motor developmental delay, neurodevelopmental delay, respiratory distress, brainstem abnormality MRI, white matter abnormality MRI, leukoencephalopathy, elevated lactate, complex IV defect	***MRPS25***	c.329+75G>A	Intronic	AE, AS, Var
					NM_022497.4	c.329+75G>A	Intronic	
**11**	**R51757**	M	Infant	Motor developmental delay, neurodevelopmental delay, seizures, feeding difficulties, elevated lactate, complex I defect	***NDUFA10***	c.-99_-75del	5′UTR	AE
					NM_004544.3	c.-99_-75del	5′UTR	
**12**	**R80346**	F	Birth	MDDS, seizures, encephalopathy, hypotonia, died as neonate, elevated lactate, complex III, IV and V defects	***LIG3***	c.86G>A	Stop	AE, Var
					NM_002311.4	c.1611+208G>A	Intronic	
**13**	**R20754**	M	Neonatal	Nystagmus, hearing impairment, white matter abnormality MRI	***UFM1***	c.-273_-271del	Promoter	AE
					NM_016617.2	c.-273_-271del	Promoter	
**14**	**R25473**	F	Adult	Usher syndrome, immune abnormality, neutropenia, abnormality retina, cataract, visual impairment, hearing impairment	***PEX1***	c.1842del	Frameshift	AE, Var
					NM_000466.2	c.1240-1551A>G	Intronic	
**15**	**R28774**	M	Infant	Myopathy, neurodevelopmental delay, hypotonia, movement disorder, failure to thrive, feeding difficulties, died as a young child due to recurrent respiratory infections, complex I defect	***TIMMDC1***	c.596+2146A>G	Intronic	AE, AS, Var
					NM_016589.3	c.596+2146A>G	Intronic	
**16**	**R96820**	F	Neonatal	Muscle weakness, neurodevelopmental delay, hypotonia, microcephaly, cardiomyopathy, hearing impairment, elevated lactate, metabolic acidosis, complex IV defect	***CLPP***	c.661G>A	Splice region	AE, AS
					NM_006012.2	c.661G>A	Splice region	
**17**	**R21147**	M	Infant	Neurodevelopmental delay, feeding difficulties, elevated lactate, complex I defect	***NDUFA10***	c.-99_-75del	5′UTR	AE
					NM_004544.3	c.-99_-75del	5′UTR	
**18**	**R64921**	M	Child	Ophthalmoplegia, speech delay, developmental regression, ataxia, abnormality retina, visual impairment, complex I defect	***MCOLN1***	c.681-19A>C	Intronic	AE, AS
					NM_020533.2	c.832C>T	Stop	
**19**	**R52016**	M	Infant	Died as infant, basal ganglia abnormality MRI, neurodevelopmental delay, encephalopathy, hypotonia, myoclonus, nystagmus, abnormality eye movement, neuropathy, brainstem abnormality MRI, elevated lactate, complex I defect	***TIMMDC1***	c.596+2146A>G	Intronic	AE, AS, Var
					NM_016589.3	c.596+2146A>G	Intronic	
**20**	**R46723**	F	Infant	Basal ganglia abnormality MRI, encephalopathy, brainstem abnormality MRI, complex I defect	***NDUFAF5***	c.2T>C	Start loss	AE, AS, Var
					NM_024120.4	c.223-907A>C	Intronic	
**21**	**R58859**	M	Adult	Ophthalmoplegia, myopathic facies, myalgia, diabetes, arrhythmias	***TAZ***	c.348C>T	Synonymous	AS
					NM_181313	c.348C>T	Synonymous	
**22**	**R80184**	M	Prenatal	Muscle weakness, myopathy, neurodevelopmental delay, intellectual disability, seizures, hypotonia, dystonia, spasticity, microcephaly, growth delay, failure to thrive, respiratory distress, cataract, abnormality eye movement, delayed myelination, hypoplasia of the corpus callosum, lack of insular opercularization, died as a young child from pneumonia, elevated lactate, complex I and I/III defects	***ALDH18A1***	c.1982C>A	Stop	AE, MAE
					NM_001017423.1	c.1858C>T	Missense	
**23**	**R59185**	F	Child	Basal ganglia abnormality MRI, ophthalmoplegia, ataxia, growth delay, arrhythmias, optic atrophy, visual impairment, neuropathy, white matter abnormality MRI, elevated lactate	***NDUFS4***	c.466_469dup	Frameshift	AE
					NM_002495.2	c.466_469dup	Frameshift	
**24**	**R63087**	M	Child	Basal ganglia abnormality MRI, muscle weakness, myopathy, rhabdomyolysis, neurodevelopmental delay, seizures, infection related deterioration, elevated lactate	***SLC25A42***	c.380+2T>A	Splice donor	AS
					NM_178526.4	c.380+2T>A	Splice donor	
**25**	**R44456**	F	Infant	MADD, respiratory distress, dysmorphic features	***MRPL44***	c.179+3A>G	Splice region	AE, AS
					NM_022915.3	c.179+3A>G	Splice region	
**26**	**R33391**	F	Infant	Failure to thrive, elevated lactate, complex I defect	***NDUFAF5***	c.605dup	Frameshift	AS, Var
					NM_024120.4	c.223-907A>C	Intronic	
**27**	**R66696**	M	Young child	Muscle weakness, myopathy, rhabdomyolysis, infection related deterioration, died as child, complex I, III and IV defects	***LPIN1***	c.2550-865_2667-34del	Deletion	AS
					NM_001261427.1	c.2550-865_2667-34del	Deletion	
**28**	**R24289**	M	Young child	Hypotonia, developmental delay, hearing impairment, white matter abnormality on MRI, lactic acidemia, hyperlactacidemia, proteinuria, glycosuria	***RRM2B***	c.328C>T	Missense	AE, MAE
					NM_015713.4	c.?	Intergenic	
**29**	**R98349**	F	Infant	Clotting defect, lactic acidosis	***DLD***	c.685G>T	Missense	AS
					NM_000108.5	c.1046+5G>T	Splice region	
**30**	**R91273**	F	Adult	MADD during pregnancy	***ETFDH***	c.687_688del	Frameshift	AE
					NM_004453.3	-		
**31**	**R60537**	M	Neonatal	Congenital disorder of glycosylation, seizures, cognitive impairment, nose abnormalities, large fleshy ears, abnormal isoelectric focusing of serum transferrin	***ATP6AP1***	c.291-135C>T	Intronic	AE, Var
					NM_00183.4	c.291-135C>T	Intronic	
**32**	**R70961**	M	Young child	Leigh syndrome, optic atrophy, parkinsonism, status epilepticus, developmental regression, abnormal thalamic size, lactic acidosis, urinary glycosaminoglycan excretion	***PTCD3***	c.1519-1G>C	Splice acceptor	AS
					NM_017952	c.1918C>G	Missense	

### Aberrant expression

**Fig. 3:**
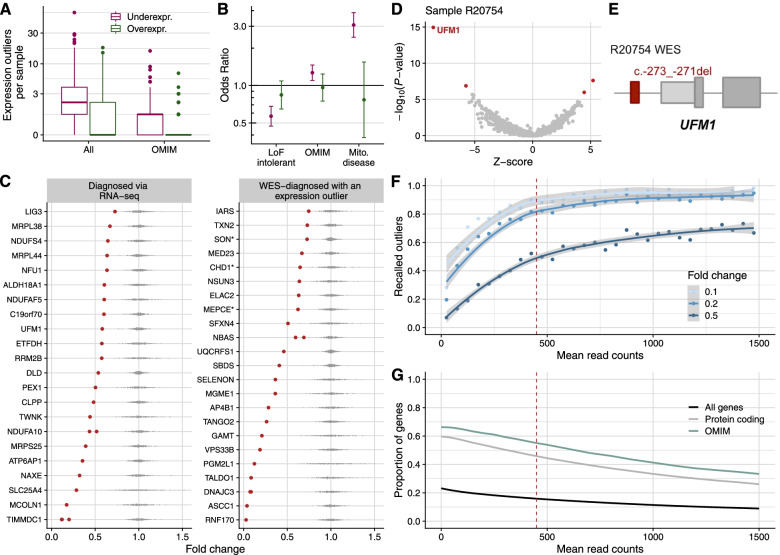
Aberrant expression. **A** Distribution of genes per sample that were detected as expression outliers, for all genes and genes known to cause a disease (OMIM), stratified by outlier class. **B** Observed over expected number of overexpression and underexpression outliers (y-axis, log-scale) for loss-of-function intolerant genes, OMIM genes, and mitochondrial disease genes (x-axis). Error bars represent 95% confidence intervals of pairwise logistic regressions. **C** Gene expression fold change relative to the OUTRIDER-modeled expected value of all disease-causal genes that were aberrantly expressed in their corresponding affected sample. Each dot corresponds to a sample, with the affected ones in red. Data stratified by cases diagnosed via RNA-seq (*n* = 25) and diagnosed via WES (*n* = 22). Genes with a dominant mode of inheritance are marked with a * (*n* = 3). The two *NDUFA10* cases are siblings, as well as the two *DNAJC3* cases. The three *TIMMDC1* cases are unrelated. **D** Gene-level significance (−log_10_(*P*), *y*-axis) versus *Z*-score, with *UFM1* labeled among the expression outliers (red dots) of sample R20754. **E** Schematic depiction of the NM_016617.2:c.-273_-271del *UFM1* deletion (red rectangle) detected by WES in sample R20754. Figure not shown at genomic scale. **F** Fraction of recalled underexpression outliers simulated with different fold changes (depicted in shades of blue) per mean gene expression (measured in raw read counts). Recall was computed in 50-wide intervals and dots are depicted in the center of the intervals. At a mean read count of 450 (vertical red dashed line), half of the simulated outliers with a fold change of 0.5 are recalled, allowing for investigating dominant genes and compound heterozygotes genes with a single downregulated allele. **G** Proportion of genes expressed at a given mean expression or higher, colored by different gene classes. Genes are taken from the GENCODE annotation, release 29 (Methods). A total of 9656 genes (16%), 9325 protein coding (46%), and 2098 OMIM genes (55%) have a mean read count higher than 450

A total of 14,100 genes were considered in the strand-specific subset and 14,399 in the non-strand-specific subset (Additional file 2: Fig. S2, Methods). In both cohorts, this represented 66% of the OMIM genes and 90% of the mitochondrial disease genes (Methods). OUTRIDER called a median of two underexpression outliers per sample, including one known disease gene, and a median of one overexpression outlier per sample, at a false discovery rate (FDR) less than 0.05 (Fig. 3A). Both over and underexpression outliers are also seen in unaffected controls, therefore outliers are not necessarily indicative of a pathological event [50]. One sample presented a considerably higher number of underexpression outliers than the rest (*N* = 61). Its sequencing depth (61 million reads) and high-quality exonic ratio (87%) were not particularly different from the rest. It was collected in a center among nine others and sequenced in a batch among 95 others, discarding a possible center or batch effect. It belongs to a neonatal mitochondrial disease patient, which is the most recurrent clinical presentation of our cohort. This patient was the only one of West Asian origin, suggesting ancestry as a potential explanation for the high number of outliers.

In agreement with observations in non-affected individuals [50, 83], we found enrichment for loss-of-function rare variants among underexpression outliers (Additional file 2: Fig. S5). Also, loss-of-function (LoF) intolerant genes were depleted for underexpression outliers (Fig. 3B), reflecting constrained expression. Given the clinical diagnosis of the individuals in our study, we observed an enrichment for OMIM genes (1.25-fold), and particularly mitochondrial disease genes (3-fold) among underexpression outliers (Fig. 3B).

For candidate gene prioritization, we focused on underexpression rather than overexpression outliers because we presumed LoF to be a more likely pathomechanism of congenital metabolic disorders than dominant-negative and gain-of-function [52]. Aberrant expression was a major contributor to our diagnostic success with 25 out of 32 (78%) newly diagnosed cases pinpointed as expression outliers (Fig. 3C). In Fig. 3D–E, we illustrate how expression outlier detection supported the identification of a causative variant in the case of a male with neonatal-onset leukodystrophy, nystagmus, and hearing impairment, whose initial WES analysis was inconclusive. RNA-seq analysis revealed two underexpression outliers, among which was the ubiquitin-fold modifier 1 gene, *UFM1* (MIM: 610553, Fig. 3D). This sample presented the lowest expression of *UFM1* among all 303 samples (Fig. 3C). Reinspection of WES revealed a 3-bp homozygous deletion located in the promoter region (NM_016617.2:c.-273_-271del, Fig. 3E), which was initially not prioritized during WES analysis due to its location. This variant has recently been reported to significantly reduce promoter and transcriptional activity and was considered to be pathogenic in cases of hypomyelinating leukodystrophy [84], thus confirming the diagnosis with *UFM1*. This case exemplifies how the detection of aberrant expression enables the reprioritization of variants located in the non-coding regions.

As in half of our solved cases, the fold change reduction was around 50% (Fig. 3C), we studied the sensitivity to detect underexpression outliers of that magnitude. We simulated outliers in all genes (in batches of 300 not to affect the global FDR estimations) with different fold changes (0.1, 0.2, and 0.5) and tested how many of them were recalled with respect to the gene mean expression. Half of the simulated outliers with drastic fold changes were detected in genes with a mean expression of 100, but for reduction of 0.5, expected by heterozygous variants with strong effect, a mean expression of 450 was needed (Fig. 3F). Overexpression outliers are less sensitive to mean expression and the majority of simulated outliers with a fold change of 2 were recovered at a mean expression of 100 read counts (Additional file 2: Fig. S6). In our cohort, with a median sequencing depth of 90 million, 46% of protein coding and 55% of OMIM genes had a mean expression greater than 450 (Fig. 3G). The dispersion of a gene, quantified as the biological coefficient of variation (BCV [85]), also plays a role in outlier detection. Using the same scheme as for mean expression, we found that most of the outliers simulated with half reduction cannot be detected in genes with an OUTRIDER-corrected BCV greater than 0.12 (Additional file 2: Fig. S7A, Methods). Among all genes, 36% protein coding and 42% OMIM genes have a mean expression greater than 450 read counts and a BCV lower than 0.12, thus allowing for heterozygous variants with a reduction or increase by half to be detected as significant (Additional file 2: Fig. S7B). For calling outliers simulated with a more drastic fold change of 10-fold, relevant to homozygous and compound heterozygous situations, we found that 50% of OMIM genes and 42% of protein-coding genes had sufficient coverage and a low enough BCV. We provide the mean expression and dispersion of each gene, as well as the fraction of recalled outliers with fold changes of 0.1 and 0.5 in Additional file 1: Table S5. Of note, over-dispersion can be due to technical, but also to biological reasons. If the expression of a gene is naturally very variable between individuals, then large fold changes are expected and are probably not disease-causing. In this respect, it is not a drawback of aberrant expression callers, but a desired feature, to not report outliers for genes whose expression is very variable in the general population.

Detection of aberrant expression can directly pinpoint the causative gene, but it can also reflect downstream effects, which provides functional evidence and can guide or support diagnostic interpretation. This is exemplified by two cases with at least 10 significantly downregulated mitochondrial DNA-encoded genes each (Additional file 2: Fig. S8). In the first case, a 3-bp homozygous deletion (NM_133259.3:c.2595_2597del, p.Val866del) was identified in the *LRPPRC* gene (MIM: 607544 [63]), encoding for a leucine-rich PPR motif-containing protein that forms a ribonucleoprotein complex with SLIRP to regulate the stability of mature mitochondrial transcripts [86]. With RNA-seq data we observed a lower abundance of mitochondrial transcripts associated with the deletion of Valine 866 and thereby confirmed the molecular diagnosis for this patient by a functional readout directly related to LRPPRC. In the second case, stop and intronic compound heterozygous variants (NM_013975.4: c.86G>A, p.Trp29*; c.1611+209G>A, p.?) were found in the *LIG3* gene (Table 1), which is critical for mitochondrial DNA integrity [87]. The stop variant further caused *LIG3* to be an expression outlier (Fig. 3B). The high number of downregulated mtDNA genes supports the functional defect of LIG3, also seen at the protein level [30]. These two vignettes indicate that pathway analysis of RNA-seq outliers can be helpful to support diagnostics. However, more systematic studies are needed to establish the utility of pathway analysis among outliers in diagnostics.

In summary, we identified a median of one OMIM underexpressed gene per case representing the pathogenic effect in 16% of all previously diagnosed and undiagnosed cases and being the most common RNA aberration. Interestingly, in 20% of aberrantly expressed cases, the causative variant was non-coding.

### Aberrant splicing

**Fig. 4:**
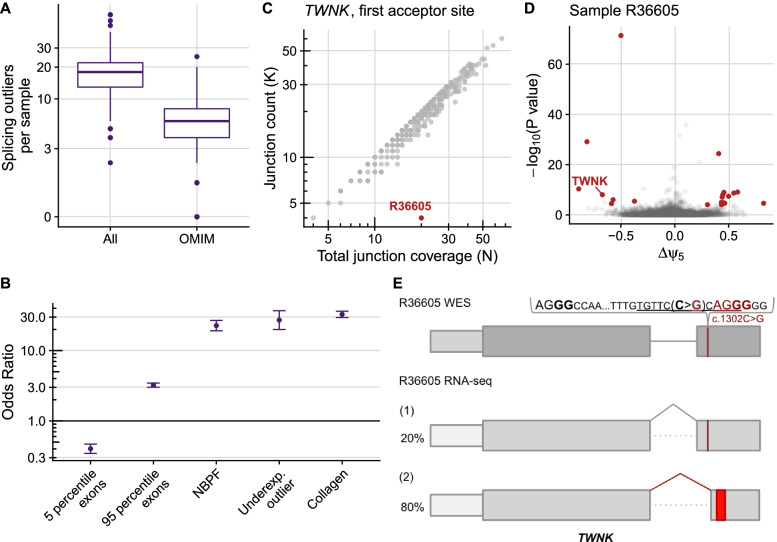
Aberrant splicing. **A** Distribution of genes per sample that had at least one splicing outlier, for all genes and genes known to cause disease (OMIM). **B** Observed over expected number of splicing outliers on different gene categories. Neuroblastoma breakpoint family (NBPF) and collagen genes were chosen due to their high number of exons and due to collagen genes alternative splicing in a developmental-stage manner and NBPF genes having a repetitive structure, which exposes them to illegitimate recombination. Error bars represent 95% confidence intervals of pairwise logistic regressions. **C** Split-read counts (*y*-axis, gray junction on panel **E**) of the first annotated junction of *TWNK* against the total split-read coverage (*x*-axis, gray and red junctions on panel **E**) of the first donor site of *TWNK*. Many samples are not exclusively using the annotated junction (scattered below the diagonal), leading to a reference 𝜓_5_ for the annotated junction of 87%. The observed 𝜓_5_ for the first acceptor site of *TWNK* in the outlier sample is 20% (obtained by dividing the junction reads by the total junction coverage, 4/20). **D** Gene-level significance (−log_10_(*P*), *y*-axis) versus differential splicing effect (observed minus expected usage proportion of the tested donor site, Δ𝜓_5_, *x*-axis) for the alternative splice donor usage in sample R36605. Gene-level significance was obtained after multiple-testing correction across junctions. Outliers are marked in red and the gene *TWNK* is explicitly labeled. The Δ𝜓_5_ value for the first donor site of *TWNK* in this sample is − 0.67 = 0.2–0.87. **E** Schematic depiction of the synonymous NM_001163812.1:c.1302C>G (p.Ser434=) *TWNK* variant and its consequence on the RNA level, activating a new acceptor site (ACGG in red) and leading to the creation of a premature termination codon (red rectangle) in sample R36605. The percentage of each transcript isoform is shown next to it. Figure not shown at genomic scale

Aberrant splicing can be caused by variants in the canonical splice sites, but also by variants in weak splice sites or in less clearly mapped splicing regulatory sequences such as the exonic and intronic splicing enhancers and silencers [88]. The aberrant splicing caller FRASER [47] is based on annotation-free intron-centric metrics [89]. FRASER uses percent spliced-in of alternative donor sites (𝜓_5_) and alternative acceptor sites (𝜓_3_) to detect exon skipping, exon creation, exon truncation, and exon elongation, in addition to splicing efficiency (𝜃) to detect intron retention (Methods). After applying FRASER, we obtained a median of 18 genes with at least one aberrantly spliced junction per sample (junction FDR < 0.1 and differential 𝜓_5_, 𝜓_3_, or 𝜃 > 0.3 and gene-wise FWER across junctions < 0.1), including 6 disease genes (Fig. 4A). Three samples had a high number of splicing outlier genes, out of which two corresponded to the ones with the highest sequencing depth. This indicates that sensitivity to splicing outliers could increase with deeper sequencing, which is in line with analyses in simulated and real datasets [29, 90]. Power analysis for FRASER has been described in the original publication on suprapubic skin tissue from the GTEx dataset, which has a similar sequencing depth to this study’s dataset. In brief, in junctions with low mean coverage (lowest third, mean junction coverage ≤ 16 reads), FRASER was able to recall 55% of simulated outliers that have a differential 𝜓 around 0.5. The recall increased to 90% in junctions with higher coverage in the analyzed dataset [47].

Aberrantly spliced genes contained significantly more rare variants than non-splicing outlier genes (Additional file 2: Fig. S9). As expected from other studies [83, 91], many of these rare variants are located in the splice region, but we also observed an enrichment of coding and intronic variants, underscoring their role in splicing. Although we controlled for multiple testing within genes [47], genes with a high number of exons (*n* > 95th percentile) had enrichment of aberrant splicing, while genes with a low number of exons (*n* < 5th percentile) showed less aberrant splicing (Fig. 4B). In particular, the neuroblastoma breakpoint family (median of 35 exons per gene) and collagen genes (median of 54 exons per gene) are more frequently aberrantly spliced, probably due to the fact that they have more exons compared to a median of 17 exons per gene detected by FRASER. In addition, splicing outliers were found to be 25-fold enriched among underexpression outliers (Fig. 4C), some of which could be explained due to the creation of an aberrantly spliced isoform containing a premature termination codon (PTC) ultimately resulting in transcript degradation by NMD.

Aberrant splicing was considered disease-causative in 18 out of 32 cases (56%), 11 of which were in combination with supportive aberrant expression data (Additional file 2: fig. S4C). In Fig. 4C–E, we showcase how aberrant splicing detection helped identify the disease-causing gene defect in a male patient with early-onset acute liver failure. In this sample, 27 aberrantly spliced genes were identified by FRASER, 12 of them on disease-causal genes. Among them was aberrant splicing and expression of *TWNK* (MIM: 606075). This gene encodes the twinkle mtDNA helicase that is critical for the efficient mtDNA replication and synthesis of the nascent D-loop strands [92]. Autosomal recessive pathogenic variants in *TWNK* are associated with disorders of mtDNA maintenance, including a hepatocerebral presentation associated with mtDNA depletion [93]. FRASER called a significant deviation from the canonical junction usage of the first intron (Fig. 4C), whereby an alternative acceptor within exon 2 was utilized, resulting in a frameshift and creation of a premature termination codon (p.416Glyfs*7, Fig. 4E), leading to aberrant expression (fold change: 0.43). Reanalysis of WES data revealed a rare homozygous variant (NM_001163812.1:c.1302C>G) in the second exon, which is predicted to have a synonymous effect (p.Ser434=). The variant is positioned four nucleotides upstream of an alternative splice junction (Fig. 4E), which corresponds to a weak splice site in controls (𝜓_5_ = 9%). This variant is predicted (using the Human Splicing Finder in silico tool [94]) to disrupt the plausible exonic splicing enhancer sequences tgttcCca and ccCagg (Fig. 4E), thereby activating the weak splice site. The activation of weak splice sites is a likely disease-causing phenomenon and is known to be recurrent, as we reported in an earlier study [23].

To assess the added value of RNA-seq over DNA sequencing only, we retrospectively analyzed the performance of two sequence-based algorithms (SpliceAI [95] and MMSplice [96]) in two ways. First, we evaluated their prediction in the 21 pathogenic aberrant splicing variants (18 from the RNA-seq-diagnosed cohort, 8 from the WES-diagnosed one, minus 5 that were either large deletions, stop, or frameshift). SpliceAI recovered 12 of these 21 variants (57%) using the recommended cutoff (SpliceAI: delta score > 0.5). MMSplice recovered only 8 (38%), using a cutoff to capture percent spliced-in psi differences of 30% (|Δlogit(Ψ)| > 1.24). For both methods, most of the recovered variants were in the splice region and performed poorly with coding and intronic variants (Additional file 2: Fig. S10A). Second, we applied these two methods genome-wide to our WGS samples (*n* = 23). This yielded a manageable number of predicted rare variants (median MMSplice = 23 and SpliceAI = 12 per sample, Additional file 2: Fig. S10B). However, only 12.5% of SpliceAI and 10% of MMSplice predicted variants in expressed genes were supported with an aberrant splicing call. Altogether, direct experimental observations of aberrant splicing by RNA-seq are still far from being accurately predicted by variant annotation tools. Moreover, besides scoring a junction, RNA-seq reveals the consequence of the splicing defect on the resulting transcript isoform (e.g., frameshift or exon truncation), which is crucial for diagnostics.

A particular value of RNA-seq lies in the quantification of different transcript isoforms. This is especially useful for transcripts with physiological presence of several alternative isoforms and in cases of aberrant splicing with a complex pattern, exemplified by a case with a homozygous splice region variant (NM_022915.3:c.179+3A>G) in the gene *MRPL44* (MIM: 611849). This variant led to transcript depletion and three alternative isoforms with a PTC in each, in addition to the main transcript isoform that was present in less than 18% of all reads (Additional file 2: Fig. S11).

Overall, a pathogenic splice defect was found in 9% of the cases. Compared to aberrant expression analysis, aberrant splicing analysis yielded nearly ten-fold more outliers per sample and less frequently led to pinpointing the causative variant. Aberrant splicing analysis was particularly useful to identify pathogenic intronic, missense, and synonymous variants.

### Mono-allelic expression

**Fig. 5:**
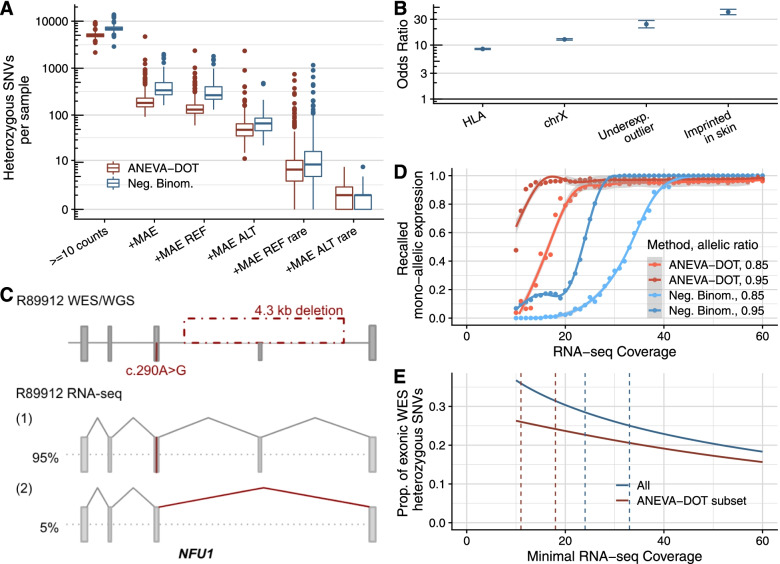
Mono-allelic expression. **A** Distribution of heterozygous SNVs per sample for successive filtering steps from left to right: Heterozygous SNVs detected by WES with an RNA-seq coverage of at least 10 reads, where MAE is detected, where MAE of the reference is detected, where MAE of the alternative is detected, and subsetted for rare variants. MAE expression detected using ANEVA-DOT and a negative binomial test (Methods). **B** Odds ratio of MAE in genes with common variants only and with at least one rare variant across different gene categories. Results shown for the negative binomial test only. Error bars represent 95% confidence intervals of pairwise logistic regressions. **C** Schematic depiction of the disease-causing 4.3 kb deletion and the c.290A>G SNV in *NFU1*, and their consequence on the RNA level in sample R89912. The percentage of each transcript isoform is shown next to it. Figure not shown at genomic scale. **D** Fraction of recalled MAE events (FDR < 0.05 on each method) with simulated allelic ratios of 0.85 and 0.95 as function of RNA-seq coverage. **E** Proportion of exonic heterozygous WES SNVs detected in all genes as a function of minimal RNA-seq coverage. ANEVA-DOT is able to detect only a subset of SNVs. Vertical lines correspond to RNA-seq coverage needed to recall 90% of simulated allelic ratios of 0.85 and 0.95 as inferred from panel D. REF: reference, ALT: alternative, rare: minor allele frequency < 0.1%

For heterozygous loci, the expression of only one of the two alleles is referred to as mono-allelic expression (MAE). Possible causes for MAE include one allele being transcriptionally silenced or post-transcriptionally degraded and can have genetic or epigenetic grounds [97, 98]. As heterozygous variants alone are discarded when investigating autosomal recessive disorders, the detection of MAE of a rare variant indicates that both alleles are affected and enables their prioritization. We call MAE on heterozygous single-nucleotide variants (SNVs) with at least 10 reads (median: 6,901 per sample, Fig. 5A) using the negative binomial test of Kremer et al. ([23], FDR < 0.05, and allelic imbalance >80%, Methods) and ANEVA-DOT ([49], FDR < 0.05). ANEVA-DOT requires an estimate of each gene expression variation per tissue, which for fibroblasts is precomputed and provided for 6364 genes, including 40% of OMIM genes. This restricts the method to 72% of heterozygous SNVs with a coverage of at least 10 reads (median of 5043 per sample, Fig. 5A). The negative binomial test called 6% of the tested variants significant and ANEVA-DOT 4.7%. We observed that MAE was more frequent towards the reference than towards the alternative allele using both methods (Fig. 5A). Subsetting to rare variants, we found a median of 6 to 8 mono-allelic expression events of the reference allele and 1 of the alternative (Fig. 5A).

As expected from their biology, MAE was enriched among HLA, X-chromosomal, and imprinted genes (when using the negative binomial test only as ANEVA-DOT does not provide an estimate for the variation in X-chromosomal genes and most imprinted genes, Fig. 5B). Moreover, we found a high enrichment of underexpression outliers (Fig. 5B), indicating that loss of expression of a single allele can be detected as an aberrant expression of the gene itself.

Detection of MAE helped to diagnose four cases, detected by both methods, all coupled with aberrant expression (Additional file 2: Fig. S4). In one case, an infant male with severe Leigh syndrome and complex I deficiency, MAE of *NFU1* (MIM: 608100) was identified (97% of the alternative allele) in combination with aberrant expression (fold change: 0.63, only outlier in *NFU1* in Fig. 3C). *NFU1* encodes a scaffold protein that facilitates the insertion of iron-sulfur clusters into the subunits of the respiratory chain complexes and lipoic acid synthase [99, 100]. Individuals harboring pathogenic biallelic *NFU1* variants present with early-onset failure to thrive, pulmonary hypertension, encephalopathy, and neurological regression [101, 102]. The mono-allelically expressed missense variant NM_001002755.2:c.290A>G (p.Val91Ala) with a CADD score of 28.6 was absent from gnomAD. Following this observation, we initiated WGS, which revealed a 4.3 kbp heterozygous deletion affecting exon 6 of the second allele, explaining the detected MAE (Fig. 5C). Proteomic analysis also found a severe reduction of NFU1 (fold change: 0.13), finally confirming the diagnosis [30]. This case presents the first association of pathogenic *NFU1* variants with Leigh syndrome, thus expanding the clinical phenotype. Interestingly, pathogenic variants in another iron-sulfur cluster scaffold gene, *BOLA3* (MIM: 613183), and in a gene involved in iron-sulfur cluster biosynthesis, *FDXR* (MIM: 103270), have previously been reported to cause Leigh syndrome [103, 104].

Haploinsufficiency has been reported as a pathomechanism for more than 660 genes [105, 106]. It appears especially important for neurodevelopmental disorders, where *de novo* variants are often found in haploinsufficient genes or regulatory non-coding regions [107, 108]. Hence, although the majority of mitochondrial diseases are inherited in an autosomal recessive mode [52], we also considered the possibility of haploinsufficiency, which can be detected by a combination of underexpression and MAE. Three samples in our cohort were solved with known haploinsufficient genes (*MEPCE* (MIM: 611478, ref. [67]), *SON* (MIM: 182465), and *CHD1* (MIM: 602118), Additional file 1: Table S4). They were all called outliers with close to 50% reduction in expression levels (fold change: 0.56, 0.61, and 0.64, respectively, Fig. 3C). Each of these genes had heterozygous protein-truncating variants suggesting that NMD acted on the transcript originating from the alternative allele. This hypothesis was confirmed by MAE of the reference alleles (88%, 91%, 75%, respectively). Moreover, segregation analysis in each case confirmed that the protein-truncating variants occurred *de novo*. Altogether, these results demonstrate that aberrant expression callers controlling for hidden confounders such as OUTRIDER [45] are sufficiently sensitive to detect aberrant expression when only one allele is affected and may discover the pathological variant in autosomal dominant disorders, particularly those presenting haploinsufficiency.

We identified MAE in nine disease-associated SNVs with a median alternative allelic ratio of 0.95. We next explored the minimum coverage of an SNV to detect MAE by simulating allelic counts. Using the negative binomial method, we found that an allelic ratio of 0.95 can be detected in more than half of the SNVs with coverage of at least 24 reads (Fig. 5D). This coverage is met by 28% of all heterozygous exonic WES SNVs from our samples (Fig. 5E). To detect an alternative allelic ratio of 0.85 in the majority of the cases, a coverage of at least 33 is needed, a coverage met by 25% of all heterozygous exonic WES SNVs. In comparison, ANEVA-DOT required lower coverage for calling MAE (11 reads for recalling allelic ratios of 0.95 and 18 reads for allelic ratios of 0.85). However, as ANEVA-DOT is limited to a subset of genes, this translated into similar proportions of variants which can be effectively analyzed as with the negative binomial test (26% for allelic ratio of 0.95 and 24% for allelic ratio of 0.85, Fig. 5D).

Altogether, ANEVA-DOT and the negative binomial appear as complementary methods, with a similar proportion of assessable variants. Both methods led to the identification of the same pathogenic variants in our cohort. ANEVA-DOT performs better at lower coverage. However, it considers only a subset of genes leaving out all X-linked and most OMIM imprinted genes which are relevant for molecular diagnostics.

### Variant calling in RNA-seq data

**Fig. 6:**
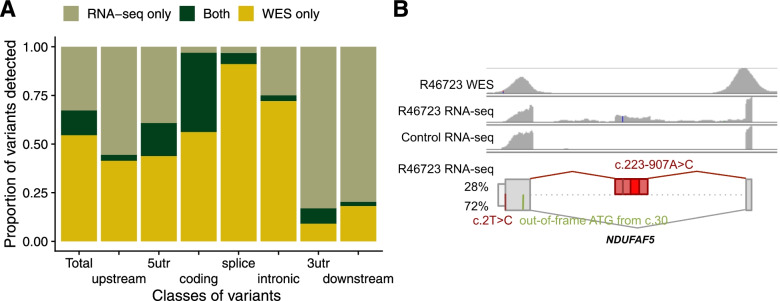
RNA-seq variant calling. **A** Median across samples of the proportion of variants called only by WES, only by RNA-seq, and by both technologies, in total and stratified by variant classes. Of note, over 50% of variants in coding regions are called only by WES, probably because of RNA-seq limitations including that not all the genes are expressed in fibroblasts, the uneven read coverage along the transcript, and because the expression level of variant-carrying alleles must be high enough to yield sufficient RNA-seq read coverage. **B** WES (row 1) and RNA-seq (row 2) coverage of the affected sample (R46723) and a representative control (row 3) using IGV. The created exon and a variant are seen in the affected RNA profile, but not covered in the corresponding WES and not present in the control. Bottom row: schematic depiction of the NM_024120.4 c.2T>C and c.223-907A>C variants and their consequence on the RNA level with an out-of-frame ATG (in green), and a cryptic exon with the PTC (bright red rectangle) on *NDUFAF5*. The percentage of detected transcript isoform is shown next to it. Figure not shown at genomic scale

Currently, the application of WGS is still emerging within a diagnostic setting, largely limiting the sequence analysis to coding variants detected by WES. Advantageously, RNA-seq is able to contribute to variant discovery [26] by covering UTRs and even, with a lesser sequencing depth, intronic regions, which are not well covered by exome-capturing kits [109]. We called variants in our RNA-Seq data following GATK’s best practices (Methods). To identify filtering criteria with a useful balance between recall and precision, we performed a benchmark using 210 RNA-seq samples derived from suprapubic skin of the GTEx project (Methods), considering their corresponding WGS-based variants as the ground truth. This benchmark suggested the exclusion of regions with three or more variants within a 35-bp window, repeat masked regions [42], and variants with less than three reads supporting the alternative allele. Filtering these variants yielded a precision of 95% (97%) and a recall of 40% (54%) for heterozygous (homozygous) variants, in genomic positions with an RNA coverage of at least three reads (Additional file 2: Fig. S12A, Methods). While a precision of 95% would not be recommended for genome-wide variant prioritization, we found it to be reasonable for variant detection in candidate genes identified by aberrant expression and splicing analyses. When applied to our rare disease cohort, these filters yielded a median of 44,154 variants per RNA-seq sample, in comparison to a median of 63,666 variants found by WES (Additional file 2: Fig. S12B), including a median of 19,252 variants not called by WES. As expected, RNA-seq was particularly helpful in revealing variants in the untranslated regions (40% RNA-seq only in 5′UTR and 75% in 3′UTR, Fig. 6A). Coverage of intronic regions increased by one third by using RNA-seq based calling, which was specifically helpful for detecting deep intronic splice-altering variants (Fig. 6A).

RNA-seq variant calling identified the causative variant in nine cases missed by WES, all deep intronic (Table 1), thereby negating in some cases the need to perform WGS. All of these cases were already candidates identified via aberrant expression and/or splicing analyses. One was a female suspected mitochondrial disease patient, presenting early in infancy with failure to thrive, complex I deficiency, and elevated lactate. FRASER detected aberrant splicing in the *NDUFAF5* gene (MIM: 612360), encoding a complex I assembly factor, highlighting a cryptic exon in intron 1, present in 28% of the transcript (Fig. 6B). This 258-nt cryptic exon is in-frame and predicted to lead to an extension of the open reading frame by 31 amino acids before encountering a PTC (Fig. 6B). RNA-seq variant calling revealed a rare intronic variant (NM_024120.4:c.223-907A>C) within the cryptic exon (Fig. 6B). This variant has recently been described in a single patient, with cDNA studies supporting the creation of a new exonic splicing enhancer and the same aberrant splicing [110]. Moreover, WES had identified an unreported start-loss heterozygous variant (NM_024120.4:c.2T>C). This variant disrupts the start codon, with the next available ATG out-of-frame at position c.30. Pathogenic variants in *NDUFAF5* have been associated with an early-onset complex I deficiency, characterized by developmental delay, failure to thrive, hypotonia, and seizures [110], in agreement with the clinical presentation of the investigated individual. This intronic variant was also found associated with the inclusion of the same cryptic exon in another unrelated RNA-seq diagnosed case from our compendium, where it is in trans with a heterozygous frameshift (NM_024120.4:c.605dup) which causes aberrant expression. Notably, variant calling in RNA-seq data fails in intergenic and intronic regions, as well as in genes that are not expressed. Thus, the increased power of WGS in calling all genetic variation is still unquestionable, though the interpretation of cumbersome WGS datasets could be streamlined through the incorporation of RNA-seq data.

### Overview of the diagnosed cases

**Fig. 7:**
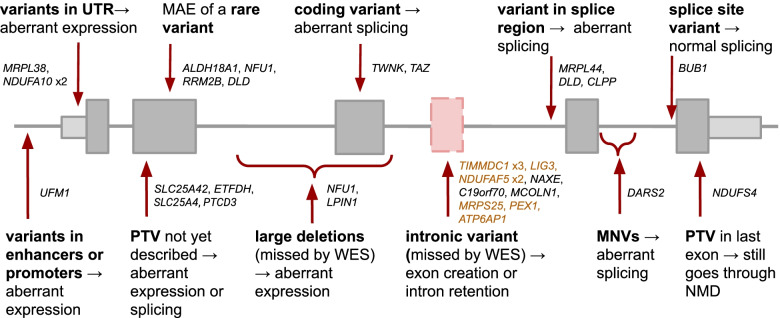
RNA-seq captures a broad spectrum of mechanisms of action of pathogenic variants. Summary of variants and their effect on transcript across 33 cases from our cohort, where the capture of a transcript event by RNA-seq enabled establishing a genetic diagnosis in 32 and rejecting a candidate gene in one case, highlighting the value of transcriptomics as a tool in diagnostics. Each gene represents one case, except for *NFU1*, which belongs to two categories. Highlighted in orange are the nine cases where the intronic variant was missed by WES but called by RNA-seq. Both large deletions were missed by WES and RNA-seq, therefore requiring WGS to be identified. PTV: protein-truncating variant. MNV: multi-nucleotide variant

In a diagnostic setting, the value of RNA-seq lies in the functional assessment of often unpredictable effects of variants, leading to their validation and (re)prioritization, or shedding light on the non-coding regions and more complex pathomechanisms. As seen from the 32 cases diagnosed using RNA-seq, this application enabled the detection of a broad spectrum of molecular pathomechanisms driven by rare variants, including aberrant expression caused by variants in the promoter, deep intronic variants generating cryptic exons, and the combined deleterious effect of two common variants in *cis* (Fig. 7).

Returning to our reference set, of the 106 WES-diagnosed cases, 32 contained at least one rare protein-truncating variant (PTV, case-by-case description in Additional file 1: Table S4). Our RNA-seq-based workflow was able to reidentify the causal gene in 84% (27 out of 32) of them. In the five remaining cases, RNA-seq failed to detect the aberrant transcripts for various reasons. In two cases, the causal genes (*CCDC40*, MIM: 613799; and *F11*, MIM: 264900) were not expressed in skin fibroblasts. Nine other disease-causal genes from our full cohort (*CYP4X1*, *GNAO1*, *GPD1*, *HMGCS2*, *KCNA2*, *MT-TL1*, *SCN1A*, *SLC7A2*, and *UNC80*) were also not expressed in fibroblasts, representing in total 10% of all disease-causal genes. In another two cases, both involving the *TXNIP* gene (MIM: 606599), the fold change was low (0.1 and 0.22), but a high dispersion of the gene’s expression led to the cases not being detected as outliers. In the last case, the patient harbored a heterozygous pathogenic missense variant in trans with a heterozygous PTV located in the first exon of the *GFER* gene (MIM: 600924) that leads to a frameshift and a PTC after 16 amino acids. The clinical presentation of the patient and his sibling (not included in our cohort) include exercise intolerance, elevated lactate, and cataracts, which matched those described in other cases harboring biallelic *GFER* variants [111], arguing in favor of pathogenicity. This case exemplifies that the functional impact of a PTC is not always explained by NMD [112].

We further inspected all rare homozygous variants and found that most of the rare stop and frameshift variants that occur outside the last exon and are expressed in fibroblasts (Methods) indeed caused an expression outlier (Additional file 2: Fig. S13A). Only one rare homozygous frameshift in the last exon led to aberrant expression, which corresponded to the *NDUFS4* case (Figs. 3C and 7), thus providing functional validation to solve the case. Similarly, most homozygous splice-site variants caused aberrant splicing (Additional file 2: Fig. S13B), with the exception of one case where normal splicing was observed (variant NM_004336.4:c.1876+2A>G in *BUB1*, Fig. 7, Additional file 2: Fig. S13C). In this respect, RNA-seq can also help to prevent a false assignment of pathogenicity. Finally, we tested the impact of all rare stop heterozygous SNVs that are not located in the last exon and found that more than 70% led to MAE of the reference allele (using the negative binomial distribution to assess all variants, Additional file 2: Fig. S13D), which was a significantly higher proportion than the rest of variant classes and is in agreement with the results obtained from GTEx [91, 113]. This highlights the need for functional validation, such as RNA-seq, for PTVs without reported pathogenicity.

Reanalysis of the cohort published in 2017 [23] provided a genetic diagnosis to an additional five cases (two *NDUFAF5*, *LPIN1*, *TAZ, NDUFA10*; Table 1). The splicing defect in *NDUFAF5* was not detected by the previously used LeafCutter method [114] but was found with FRASER. LeafCutter did find aberrant splicing in the *LPIN1* and *TAZ* genes, but no variants were found to be conclusive. For the *LPIN1* case, the causative variant (a 1,759 bp deletion) was detected in a follow-up study via WGS. For the *TAZ* case, the causative variant was initially not prioritized because of its predicted consequence [47]. In the *NDUFA10* case, the previously used method to detect aberrant expression, DESeq2 [115], did find it to be an expression outlier, but the homozygous causal variants in the 5′UTR were not initially prioritized until later when the same aberrant expression was identified in an affected sibling (two outlier samples in *NDUFA10* in Fig. 3C). This shows the importance of data reanalysis by considering updates of the disease course and family segregation, applying state-of-the-art methods, and follow-up studies.

### Tissue-specific gene expression

**Fig. 8:**
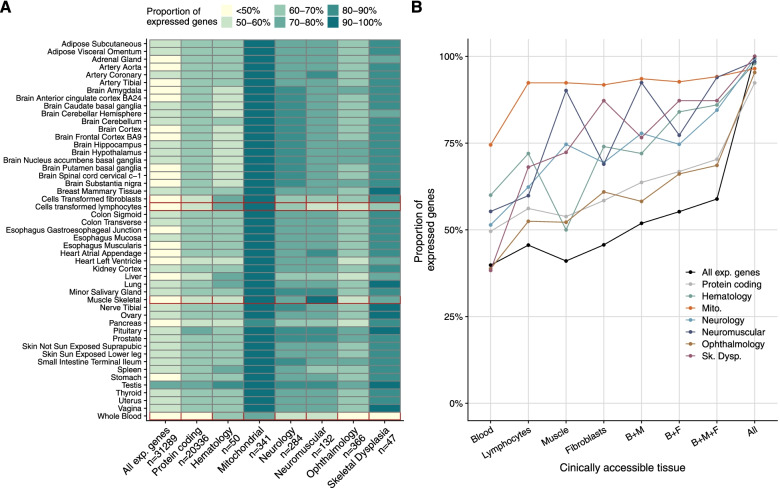
Tissue-specific gene expression. **A** Proportion of expressed genes from different categories across 49 GTEx tissues with the CATs delineated in red. **B** Proportion of expressed genes from different categories across CATs from GTEx, alone or in combination with another CAT. “All” refers to all the 49 tissues, not just the CATs. B: blood, M: muscle: F: fibroblasts, CAT: clinically accessible tissue

An important limitation of the application of RNA-seq in a clinical setting is that the causal gene may not be expressed in the sampled tissue. To assess the impact of source material on the transcriptome, we compared the expression of disease-associated genes for major disease categories (Fig. 8, Methods) across 49 tissues from healthy donors from the GTEx Consortium (Methods). The majority of genes of each disease category are expressed in any given tissue (except for ophthalmology and skeletal dysplasia genes in whole blood, Fig. 8A). Exceptionally, mitochondrial disease genes are ubiquitously expressed in all tissues, but other disease genes have a more pronounced tissue-specific expression profile, such as neurological genes in the brain (Fig. 8A). As in clinical practice biopsy of the least invasive tissue is desirable, we next focused on the clinically accessible tissues (CATs)—whole blood, Epstein-Barr virus (EBV)-transformed lymphocytes, skeletal muscle, and skin-derived fibroblasts [116]. Fibroblasts were the CAT expressing the highest number of Mendelian disease genes (2564; 67%; Fig. 8B). Although obtaining a skin biopsy is more invasive, skin-derived fibroblasts appear as a more useful resource than blood, showing a higher number of expressed genes in each disease category (which is significant for OMIM, neurology, ophthalmology, and skeletal dysplasia, Fisher’s test *P* < 0.05). Fewer genes are also expressed in muscle for all disorders, except for the neurology and neuromuscular disorders, confirming its utility for these disorders (Fig. 8B). Overall, thoughtful disease-specific selection of the biosample is increasing the sensitivity of RNA-seq-based diagnostics. Among the biosamples, fibroblast cell lines represent a good compromise between the number of expressed genes and the invasiveness of the sampling procedure. Moreover, fibroblast cell lines allow functional follow-up studies.

## Discussion

The clinical significance of any given genetic variant falls along a gradient, ranging from those in which the variant is almost certainly pathogenic for a disorder to those that are almost certainly benign [21]. The clinical interpretation of variants is based on cumulative evidence from population-wide frequencies, computational predictions, functional data, and segregation patterns. Findings from RNA-seq contribute evidence and may validate predicted pathogenic or likely pathogenic variants, reclassify VUS or benign variants, and lead to the detection of variants undetected by WES or overlooked by WES data inspection. Specifically, the value of RNA-seq is to provide functional evidence on variants affecting gene expression and splicing. Loss-of-function variation in disease genes represents the strongest evidence for pathogenicity. RNA-seq allows the detection of non-coding loss-of-function variation, including splice defects leading to the non-functional mRNA isoforms and variants abolishing transcription, which remains a challenge to predict from DNA sequence alone. Moreover, RNA-seq allows the validation or invalidation of (computational) predictions including splice-site dinucleotide and NMD by providing quantitative measures of the actual fraction of affected transcripts. In this respect, RNA-seq could become the first step in the systematic inclusion of functional data together with genetic and phenotypic information which can relatively easily be implemented in the diagnostic workflow. In addition, splice defects uncovered by RNA-seq can potentially be targeted by oligo-based therapies for which there is an increasing number of precedents (e.g., in Duchenne muscular dystrophy [117], amyotrophic lateral sclerosis—ALS [118], and an N-of-1 study of milasen in a neurodegenerative patient [119]).

RNA-seq relies on taking additional patient biopsies in addition to a sample for DNA extraction and requires early consideration in the diagnostic process. Specifically, for severe life-threatening diseases with fast progression, we recommend establishing skin biopsies in the routine process in parallel to genome-based diagnostics. As we demonstrate, fibroblast cell lines express the majority of OMIM disease genes. Moreover, fibroblast cell lines can be differentiated into other cell types to more closely reflect the disease-affected tissue [26] or into pluripotent stem cells, where as many as 27,046 genes are expressed [120, 121]. Importantly, patient-derived cell lines not only allow functional studies of the disease of the patient but also provide a DNA resource for emerging sequencing technologies such as long-read sequencing [122]. One limitation of cell lines, in contrast to direct biopsies such as whole blood samples, is the time and effort needed for their growth. Therefore, in urgent situations (e.g., neonatal cases), blood sampling is preferred as it can be processed immediately.

There have been concerns regarding the use of a biological material that does not represent the affected tissue. One concern is that the gene or its relevant isoform may not be expressed in the tissue of choice. This issue might be particularly relevant for genes with highly specific spatiotemporal expression, which can include genes implicated in developmental disorders [123]. Resources such as GTEx, Panel Analysis of Gene Expression (PAGE [26]), or MAJIQ-CAT [116] allow checking the expression of candidate genes and isoforms in clinically accessible tissues and cell types. Here, using GTEx, we demonstrate skin-derived fibroblasts to capture the majority of disease genes for major disease categories. Moreover, so long as the potential causal genes are expressed, non-affected tissues have the advantage that the transcriptome-wide consequences of the diseases are limited and hence the causal defects can more clearly stand out. Another concern is that pathogenic variants affect tissue-specific regulatory elements such as transcription factor binding sites or binding sites of tissue-specific splicing factors. However, strong regulatory effects, which one could expect for monogenic disorders, may rather be constitutive. This is the case for NMD [113]. Also, a minor proportion of variants found in GTEx associated with splicing show tissue-specific effects [124].

The sample with the highest number of expression outliers was the only one of West Asian ancestry. Similar to the genome, the transcriptome seems to be variable across ancestries [125]. Sequencing more individuals of non-European ancestry will be beneficial as it can help to distinguish between aberrant and ancestry-specific gene expression and splicing.

With the increasing adoption of RNA-seq in Mendelian disease diagnostics, we foresee the need for extended clinical guidelines, akin to the update of the ACMG/AMP guidelines necessitated by the uptake of WES as a standard diagnostic approach [21]. These extended guidelines will need a concerted discussion across the community, regarding effect sizes and statistical cutoffs to define a pathological expression phenotype. Moreover, community-accepted criteria will be needed to assign the likelihood of pathogenicity for genomic variants, integrating RNA-seq-based evidence with current features including annotations of genetic variants, computational predictions, frequency, and segregation patterns.

## Conclusions

We reported the outcome of RNA-seq implementation as part of routine diagnostics for Mendelian diseases alongside WES in our center for more than 300 individuals. We demonstrated the application of the automated computational workflow DROP, showcased detailed diagnostics successes including instances of dominant mode of inheritance, and provided a diagnostic decision workflow integrating WES, WGS, and RNA-seq. The computational analysis time for RNA-seq is comparable to the genome pipelines, typically requiring less than a week from sample preparation to reported results. RNA-seq is based on the same technology as WES/WGS, which is another favorable feature to consider when deciding to expand the diagnostic spectrum beyond the DNA sequence. Stringent *p*-value-driven results yielded a manageable number of OMIM genes with aberrant RNA events (median = 8), similar to the average number of biallelic rare non-synonymous variants inspected during diagnostic WES analysis of autosomal recessive disorders. In this study, cumulative evidence from WES and RNA-seq supported the genetic diagnosis in 16% of WES-inconclusive cases. This number falls within the range of other RNA-seq studies with unrestricted inclusion criteria ranging from 7.5 to 18% (Additional file 2: Fig. S14), and a hypothetical yield of 13.5% after retrospectively analyzing a cohort of WES-diagnosed patients [126], thereby reflecting the likely expected additional value of RNA-seq as a complement to WES. Altogether, we foresee that our streamlined experimental and computational processes will help accelerate the implementation of RNA-seq in routine diagnostics.

## Supplementary Information

**Additional file 1: Table S1.** Sample annotation. **Table S2.** Extended summary of RNA-seq diagnosed cases. **Table S3.** Summary of candidate genes pinpointed via RNA-seq. **Table S4.** Summary of WES-diagnosed cases with an RNA-defect. **Table S5.** Recalled expression outliers at different mean and dispersion.

**Additional file 2: Fig. S1.** Overview of the study. **Fig. S2.** Quality control. **Fig. S3.** DNA-RNA sample matching. **Fig. S4.** Aberrant events per sample. **Fig. S5.** Rare variants among expression outliers. **Fig. S6.** Power analysis of overexpression outliers. **Fig. S7.** Power analysis of underexpression outliers with respect to biological coefficient of variation. **Fig. S8.** Cases with many mtDNA expression outliers. **Fig. S9.** Rare variants among splicing outliers. **Fig. S10.** Splicing prediction algorithms evaluation. **Fig. S11.** Complex pattern of aberrant splicing. **Fig. S12.** Analysis of variants called by RNA-seq. **Fig. S13.** Rare variants leading to outliers. **Fig. S14.** Diagnostic rate across cohorts.

## Data Availability

Our ethics approval and consent agreements allow us to share non-identifiable patient data and analysis data only, as such, we cannot provide BAM or VCF files. The analysis data provided are the gene expression count matrices, as well as the privacy-preserving count matrices of split and unsplit reads overlapping annotated splice sites from RNA-seq. They are available for download without restriction in the Zenodo repository, independently for the non-strand-specific (https://zenodo.org/record/4646823 [79]) and the strand-specific datasets (https://zenodo.org/record/4646827 [80]). The data is made available for medical research; financial interests cannot be pursued. The data and the code to reproduce the main figures of this study are available in GitHub (https://github.com/gagneurlab/RNA_diagnostics_paper_figures [127]). The pipeline to align and call WES/WGS variants is available in GitHub https://github.com/mri-ihg/ngs_pipeline/, as well as DROP (https://github.com/gagneurlab/drop [29]). Both pipelines were previously available and not developed exclusively for this study.

## Abbreviations

CAT: Clinically accessible tissue
EBV: Epstein-Barr virus
FDR: False discovery rate
LoF: Loss-of-function
MAE: Mono-allelic expression
MNV: Multi-nucleotide variant
mtDNA: Mitochondrial DNA
NBPF: Neuroblastoma breakpoint family
NMD: Nonsense-mediated decay
PTC: Premature termination codon
PTV: Protein-truncating variant
RNA-seq: RNA sequencing
SNV: Single-nucleotide variant
UTR: Untranslated region
VEP: Variant effect predictor
VUS: Variant of uncertain significance
WES: Whole exome sequencing
WGS: Whole genome sequencing
